# Supplementary material for: Are carbon emissions trading and green financial instruments synergistic? -Comprehensive quantitative research based on content analysis
Source: PLoS One. 2024 Mar 7;19(3):e0298601. doi: 10.1371/journal.pone.0298601 (PMC10919601; doi:10.1371/journal.pone.0298601)
Supplement: S1 Data — (ZIP) [file pone.0298601.s001.zip › Data/地级市Z_F工作报告.zip!/╣π╬≈/╟╒╓▌╩╨/└┤╫╘╣1⁄2╓┌║┼í╛┬φ┐╦╩2╛▌═°í┐_2012.docx]

各位代表：

现在，我代表钦州市人民政府向大会作工作报告，请予审议，并请各位政协委员和其他列席人员提出意见。

一、克难攻坚，“十二五”实现良好开局

2011年是我市实现大发展、大跨越的一年。在市委的坚强领导下，全市上下坚持以科学发展观为统领，以“建大港、兴产业、造新城、强科教、惠民生”为发展方略，凝心聚力，排难奋进，在北部湾经济区开放开发的大潮中，迈进了工业总产值超千亿元、财政总收入超百亿元地市行列，地区生产总值、规模以上工业总产值、规模以上工业增加值、财政收入、外贸进出口五项指标增速居全区第一；中外两国政府合作共建的第三个园区（中马钦州产业园区）落户钦州，钦南台湾农民创业园获国家批准，茅尾海获批为全国第一批国家级海洋公园，五皇山获批为国家地质公园，广西第十二届运动会成功举办，北部湾大学正式开工建设，汽车、修造船、飞机项目引进取得重大突破。市三届人大七次会议确定的各项目标任务圆满完成。这些令钦州人民自豪和振奋的成就，进一步夯实了钦州的发展基础、增强了发展后劲、激发了发展活力，标志着我市在推进“五年跨越、十年巨变”，全面建设小康社会的征程中迈出了坚实步伐。钦州充满着生机与活力！

一年来，我们的主要措施是：

——坚持做大总量与提升质量并举，增长动力与发展后劲显著增强。抢抓中国－东盟自贸区建成与北部湾经济区开放开发两大国家战略的叠加机遇，注重速度和质量相结合，推动经济发展方式转变和经济结构调整，实现经济扩量、提质上档。

经济总量再上新台阶。地区生产总值突破700亿达到734.4亿元，增长22.5%；工业总产值突破1000亿达到1093亿元，增长116.7%；财政收入突破100亿达到123.14亿元，增长110.94%；外贸进出口总额29.8亿美元，增长127.5%；完成全社会固定资产投资557.8亿元，增长26.5%。

经济结构转型优化。工业经济集聚壮大。中石油1000万吨炼油厂顺产达产，完成产值554.3亿元，税收75.5亿元。中粮120万吨油脂加工、玉柴溶剂油等重大产业项目建成投产，金桂纸机、泰兴石化、钛白粉、锐丰钒钛铁等项目加快建设，新开工中石油含硫原油加工配套工程、清华同方数字电视等项目。实施“千百亿产业崛起工程”、“县域工业突破工程”、“高新产业培育工程”成效显著。规模以上工业总产值突破900亿元，达到930亿元，增加值291亿元，增长91.4%；亿元企业达到75家，新增28家，其中县域亿元企业60家，同比增加26家。三次产业结构由上年的25.4：42.0：32.6调整为21.2：52.9：25.9。其中，工业占gdp的比重从36.1%提升到47.7%。

现代农业有效推进。实施“特色农业提升工程”，荔枝、香蕉、火龙果、黄瓜皮、奶水牛、大蚝、名贵龟鳖、速丰林等八大特色优势农业在基地化产业化标准化方面取得积极进展，农业标准化基地超200万亩。推广高接换种、作物套种等技术，低产果园、密蔽果园改造成效显著。新增市级以上重点龙头企业10家。稳定粮食播种面积，粮食产量103.71万吨；销售网络不断健全与完善，大宗农产品顺产顺销。农产品质量安全和重大动物疫病防控进一步强化。造林绿化和林下经济加快发展，浦北成为全国林下经济发展示范点和全国养蜂示范县。农村道路、水利等农业和农村基础设施建设明显改善，我市成为全国唯一的所有县区均列入中央财政小型农田水利建设重点计划的地市，完成渠道防渗270公里，新增恢复灌溉面积4.19万亩，除险加固病险水库35座，解决农村30多万人口饮水安全问题。

现代服务业加快发展。年年丰广场、金湾步行街等核心商圈加快构建，世界500强零售巨头沃尔玛、广西商业品牌梦之岛百货等国内外一线品牌入驻钦州。推动宾馆升星改造，新增12家三星级以上酒店。城乡市场进一步繁荣，全社会消费品零售总额204.3亿元，增长18.6%。“引银入钦”再结硕果，中信银行入驻营业，金融机构存款余额531.8亿元、贷款余额369.4亿元，分别增长12.2%和14.9%。游客接待人数、旅游总收入分别增长21.5％和48.3%。

经济质量持续向好。人均gdp突破2万元，达到22926元，比上年增加6505元。财政收入占gdp的比重由上年的11.2%提高到16.8%。税收收入占财政收入的比重由上年的86.1%提高到92.3%。人均可用财力比上年增长8.85%。规模以上工业实现主营业务收入921.86亿元，增长141.5%，工业经济效益综合指数为430.78，比上年提高155.46个百分点。城镇居民可支配收入19248元、增长 11%，农民人均纯收入6166.8元、增长15.5%。

——坚持建大港与造新城并举，城市竞争力和城市承载力明显提升。以港口为龙头，城市为依托，充分发挥港口核心资源优势，努力推动港城联动发展，相得益彰，不断提升区域综合竞争力。

港口吞吐能力突破7000万吨。保税港区一期开港运营，整车进口口岸通过国家验收对外开放，45家企业落户保税港区，国际酒类交易中心、进口汽车检测线、恒湘物流仓储基本建成，园区产值完成186亿元。港口加速向深水化、大型化推进，建成3-8#泊位等一批10万吨级泊位和散货码头，30万吨级进港航道及油码头建设加快，开展5亿吨大港规划研究。港口吞吐能力7088万吨，吞吐量4716万吨，增长56.1%，集装箱吞吐量达40.2万标箱，增长60.2%，居北部湾之首，首次超过湛江港，北部湾集装箱干线港初具雏形。

港口集疏运体系加快形成。钦州至崇左、六景至钦州港高速公路、钦州港至大榄坪铁路基本建成，南宁至钦州高速铁路全面铺轨，龙门跨海大桥、贵（港）合（浦）高速公路开工，钦州至北海和钦州至防城港高速铁路、黎塘至钦州铁路复线改造、大榄坪至保税港区铁路加快推进。继续采取财政补贴办法鼓励航运公司扩大集装箱运输，开通至香港、越南海防等国际航线和至天津、广州、深圳等国内航线，定期航班增加到每周5班，开通了保税港至昆明的海铁联运，至昆明的定期班列每周2班。港口物流平台加快构建，开展大通关百分百满意工程，口岸服务进一步提升。完成区域性国际航运中心、综合物流加工区规划，开工建设中国—东盟国际商贸城、中国—东盟农产品大市场等一批物流专业市场。

新城建设彰显滨海特色。建成市体育中心六大场馆及周边配套设施和一批上规模上档次的商住小区，河东新区展现新风貌。滨海新城加快推进，五星级北部湾国际大酒店、白石湖公园、钻石海岸国际海鲜城、东盟生态园等一批功能性项目进展顺利，实施茅尾海综合整治一期工程。钦州港行政商务中心加快推进。“一环六横六纵七通道”路网加快建设，建成北部湾大道一期、环城西路、子材大桥等一批城区主干道路。以深化“城乡清洁工程”为抓手，以精品线路、绿化美化亮化和市容整治为重点，实施“园林生活十年计划”和“迎区运十大工程”，加大城市供水、供电、交通、污水垃圾处理等基础设施建设，新增181辆市区公交车，建成市第二水厂、钦州港水厂扩建工程、胜科污水处理厂等，中心城区绿化覆盖率达34.13%，人均公园绿地面积达7.05平方米。县城和城镇建设快速推进，城市化率37.5%，比上年提高2个百分点。钦州入选“2011年中国十佳绿色城市”，荣获广西卫生城、园林城。

——坚持发挥优势与扩大开放并举，区域合作新高地建设取得新进展。充分发挥独特的区位和政策资源优势，进一步扩大开放，深化合作，激发内力，借助外力，形成合力，努力推动互利发展。

合作平台进一步拓展。中马钦州产业园区获两国总理见证签约并揭牌，园区总体规划和控制性详规已完成中期成果，7.87平方公里启动区已完成3000亩土地征拆平整，开工建设中马大街等基础设施。钦南台湾农民创业园已引进企业3家。湖南临港产业园等一批合作园区加快规划建设。石化产业园、综合物流加工区、河东工业园区、进口资源加工区及县区工业园区进一步发展壮大。对外贸易全区增长最快，其中进口21.1亿美元，增长113.8%；出口8.7亿美元，增长168.8%。钦州成为发展平台最全、最优的城市之一。

招商引资再攀新高。围绕引进战略合作伙伴和优质项目，专业招商与产业链招商相结合，积极参与第八届中国-东盟博览会等重大招商活动，成功引进浙江翔盛粘胶纤维、华汇镍冶炼、青岛九联等重大产业项目，汽车、修造船、工程机械等装备制造业项目引进取得重大进展，与天津滨海、中交集团、光大集团、太平洋建设集团等大企业开展战略合作。新签内资项目419个，其中超亿元以上项目85个，实际引进到位区外资金439亿元，增长27.8%；实际利用外资4.1亿美元，增长29.3%。

机制体制改革取得新成果。开展城乡建设用地增减挂钩、土地“征转分离” 和海域使用权招拍挂试点，获得自治区1021公顷的用地指标追加和奖励。加强与金融机构对接，开展首笔跨境贸易人民币结算业务，成功发行信托理财产品8.4亿元，有效防范政府财政风险。进一步简政放权，将一批行政管理职能授权给钦州保税港区、钦州港经济技术开发区。扩权强县工作扎实推进。全面完成集体林权主体改革。医药卫生体制改革顺利推进，基本完成基层医疗机构综合改革三批试点，基层医疗卫生机构100%实施了国家基本药物制度，卫生事业单位绩效工资改革全面启动。征地搬迁公寓式安置逐步推进。

——坚持改善民生与共建和谐并举，社会事业全面进步。把民生改善作为发展的根本出发点，坚持富民优先、和谐共建，在就业创业、社会保障、文化教育惠民等方面加大力度，促进经济社会协调发展。

就业创业环境得到改善。深入实施“三业”行动富民工程，发放小额担保贴息贷款3.6亿元,帮助8600名农民工、被征地农民、水库移民等实现创业。农村劳动力转移、下岗失业再就业等技能培训3.4万人次，新发展微型企业195家，城镇新增就业2.57万人，农村劳动力转移就业7.29万人，城镇登记失业率2.99%。

社会保障水平不断提高。投入72.14亿元改善和保障民生，比上年增加18.48亿元。为民办实事工程超额完成，累计投入32.8亿元，超过年度计划3.5亿元。应急管理体系不断完善，突发公共事件应急处置和救援能力进一步增强。社会保障覆盖面不断扩大。基本养老保险、失业保险、城镇基本医疗保险、工伤保险、生育保险参保人数超额完成年度任务，建立健全失地农民失海渔民保障制度。解决城镇中低收入群体住房难问题，建成保障性住房5003套，开工保障性住房10071套，发放保障住房补贴849万元。

社会各项事业协调发展。教育质量不断提升，教育惠民不断加大。开工建设北部湾大学，恢复重建市第四中学。建成浦北县职教中心，职教攻坚工作通过自治区评估验收。实施学前教育三年行动计划，开工建设一批幼儿园项目。实施义务教育薄弱学校改造计划，完成107个中小学校舍安全工程，新建校舍11.5万平方米，重点面向代课人员招聘670名农村中小学教师，公开招聘1080名农村中小学特岗教师，公开招聘市区学校教师410名。成立高新技术创业服务中心，鼓励企业增强自主创新能力，全市获得专利授权174件、增长59.6%。“2+6”人才体系加快构建，在全区率先出台领军型人才引进政策，聘请50名国内外知名咨询专家，引进145名硕士以上学位人才，继续实行政府雇员制度。文化产业发展、群众性文化活动取得新成绩，广播影视事业进一步拓展。公共卫生服务体系建设加快，新建959个标准化村卫生室，新农合参合率达94.1%。节能减排成效显著，单位gdp能耗下降1%左右，主要污染物排放总量完成自治区下达的任务，“十一五”节能工作终期目标考核被评定为“优秀”等级。城镇集中式饮用水源地水质达标率100%，城市空气质量优良率99.6%。成功承办“第四届中国绿色发展高层论坛”。全市62.33%的村（社区）开展了诚信计生活动，建设226个村级人口计生服务室、新家庭文化屋，人口自然增长率9.33‰，钦北区计生工作获“国优”先进县区。加强和创新社会管理，开展“携手共创平安和谐家园”活动，推进城区复合型警务机制改革，社会矛盾纠纷大调解机制和社会治安防控体系进一步完善；坚持市领导每月定期接访群众工作制度，完善12345市长热线，畅通群众诉求渠道，切实维护群众合法权益；食品药品监督管理进一步加强，安全生产形势保持稳定。钦州荣获“建设平安广西活动先进市”荣誉称号。稳控物价，居民消费价格涨幅5.4%。海关、出入境检验检疫、海事、边检、口岸以及驻北京联络处等在钦州对外开放中发挥了重要作用；税务、人民银行、银监、工商、供电、烟草、通信、邮政、盐务等在服务地方发展中作出了贡献；民族、宗教、外事、侨务、统计、审计、农机、供销、气象、地震、水文、人防、海防、新闻出版、科普、档案、方志、妇女儿童、老龄、残疾人等事业进一步加强。

各位代表，我们举全市之力，以一流的场馆设施、一流的组织服务、一流的竞赛成绩、一流的城市形象，成功承办了广西历史上规模最大、项目最全、参赛人数最多的体育盛会，得到社会各界高度评价，国家体育总局领导赞誉开幕式“体现了钦州特色、广西风采、国家水平”。广西第十二届运动会的成功举办，推动了群众性体育活动的蓬勃开展，激发出的团结拼搏、争创一流的进取精神，将成为激励全市上下加速钦州率先崛起的不竭动力!

——坚持鼓励创新与强化执行力并举，发展环境不断优化。坚持以创新为先导，积极推动政府工作创新与绩效管理相统一，切实增强政府的创造力、执行力和公信力，创优发展环境。

创新工作成效显著。深入开展创新年活动，建立健全鼓励创新的工作机制，激发全市各级各部门在优化工作机制、简化办事程序、形成工作合力等方面有新突破。市发改委制定实施投资项目预评估和后评价制度，对投资项目进行全过程监管。市国土资源局对片区征地采取“整体征收、整体拆迁、整体安置、整体保障、整体转非”的办法，有效保障了重点项目用地。市农业局创新土地流转、金融支农方式，取得较好成效。市司法局开展“争当人民调解能手”、“法律援助零距离服务圈”等主题活动，创新司法服务，提供法律保障。市公安局设立复合型警务站，创新警务服务模式，提高了群众安全感。创新工作成为提高执行力，推动各项工作取得新成效的助动力。

效能建设不断提升。深入开展“好干部善执行”主题活动，围绕“六要六不要”强化机关作风效能建设和行政效能监察。成立重大项目建设指挥部，建立和完善重点工作定期督查、重点工程现场办公、重点问题专题协调等制度。继续推行“阳光和效能重点建设岗位”制度和平衡计分卡绩效考评制度。简化行政审批程序和办事流程，市政务服务中心办结行政审批事项20多万件，承诺提速57.4%，实际办理提速89.7%，承诺件平均办结时间为0.87个工作日，群众评议率99.5%，满意率100%，行政效能全年综合指标排名全区第二位。

依法行政全面推进。主动向人大常委会报告、向政协通报重大事项。高度重视并切实抓好人大代表议案、建议和政协委员提案的办理工作，办理人大代表建议129件，政协委员提案213件，答复率、基本满意率均达100%以上。推行重大问题集体决策、专家咨询、社会公示和社会听证等制度，扩大政务公开，科学民主决策水平不断提高。

廉政建设不断加强。认真落实党风廉政建设责任制和反腐倡廉工作部署，广泛开展廉政教育系列活动，政府工作人员的廉洁自律意识进一步增强。加强重点领域、重点部门和重点项目资金使用的审计监督，深入开展工程建设领域突出问题专项治理和商业贿赂治理，切实纠正损害群众利益的不正之风，树立了政府廉洁务实的形象。

各位代表，回顾过去一年的工作，我们欣喜地看到钦州的发展步伐更加稳健，城乡环境更加优美，百姓生活更加殷实，社会建设更加和谐。这些成绩的取得，得益于市委的正确领导和市人大常委会、市政协的有效监督和大力支持，凝聚着广大创业者、劳动者、建设者的辛劳和智慧。在此，我代表市人民政府，向全市各族人民，各民主党派、工商联、人民团体和社会各界人士，向驻钦中直区直单位、驻钦部队、武警官兵，向所有关心支持钦州建设的海内外朋友，致以崇高的敬意和衷心的感谢！

同时，我们也清醒地认识到发展中存在的困难和问题：经济总量仍然偏小，地方可用财力严重滞后于财政收入的增长。用地指标有限和征地拆迁难的制约以及融资形势依然严峻，保持投资较快增长的难度加大。节能降耗、控污减排的任务艰巨。维护社会稳定压力大。一些工作人员的公仆意识、责任意识和服务意识与人民群众的要求还有差距。对此，我们要增强忧患意识、发展意识，采取更加有力的措施加以解决。

二、振奋精神，理清思路，明确2012年工作目标与任务

2012年，是我市加快科学发展、推进“五年跨越、十年巨变”的重要一年。今年国内外经济形势总体上有利于我市加快发展。尽管国际形势复杂多变，但中国与东盟的合作、泛北部湾的合作、大湄公河次区域的合作继续深化、务实推进，有利于我们发挥区位优势，扩大开放，利用好国外特别是东盟的资源、资金、市场。尽管国内的宏观经济形势依然严峻，增长下行与物价上涨的压力以及区内资金、用地、供电偏紧的压力仍未缓解，但随着广西经济总量进入全国万亿元俱乐部的行列，城镇化、工业化步伐加快，西部大开发、北部湾经济区开发开放的政策效应与作用的凸显以及我市经济总量与质量的快速提升、干部群众信心的提振，有利于我市发挥保税港区、整车进口口岸、中马钦州产业园区等国家级平台作用，形成洼地效应，加快发展。我们必须在自治区党委、自治区人民政府和市委的坚强领导下，正确把握国内外发展带来的挑战和机遇，充分发挥我市在中国－东盟自由贸易区建成、北部湾经济区开放开发不断深入的背景下独特的区位优势、港口优势、政策资源优势，全力以赴加快赶超发展，巩固和发展好来之不易的良好态势。

今年政府工作的总体要求是：以邓小平理论和“三个代表”重要思想为指导，深入贯彻落实科学发展观，紧紧围绕实现“富民强桂”新跨越和“五年跨越、十年巨变”奋斗目标，全面实施“建大港、兴产业、造新城、强科教、惠民生”发展方略，大力弘扬“自强实干，融和共赢”的新时期钦州精神，坚持创新、实干、开放、惠民、高效、廉政，坚持稳中求进，好中求快，多出成效，深入推进经济结构战略性调整，加快工业化、城镇化和农业现代化步伐，着力推进城乡统筹发展，着力深化改革扩大开放，着力保障和改善民生，实现经济快速发展，社会和谐稳定，以优异成绩迎接党的十八大胜利召开。

经济和社会发展的主要预期目标是：生产总值增长12%；财政收入增长10%以上，全社会固定资产投资增长25%以上；规模以上工业总产值增长25%；港口吞吐量5600万吨；外贸进出口总额增长15%，其中出口增长11%；实际利用外资增长10%，引进国内到位资金增长10%；社会消费品零售总额增长18%；城镇居民人均可支配收入增长11%，农民人均纯收入增长12%；居民消费价格上涨控制在4%左右；万元生产总值能耗、化学需氧量、二氧化硫、氨氮化物和氮氧化物排放总量控制在自治区下达的目标之内；城镇登记失业率3.8%以内，城镇化率达到41%左右，人口自然增长率控制在10‰以内。

各位代表，形势的发展令人鼓舞，我们一定要抢抓机遇、乘势而上，咬紧目标、奋发进取，全面完成各项目标任务，努力发展更强的经济、建设更美的城市、创造更好的生活!

三、千方百计抓住经济总量迅速扩张的机遇，实现稳中求进，好中求快，多出成效

今年是钦州撤地设市18周年和建港20周年。回首过去，我市工业总产值由1993年的52.7亿元到2011年突破千亿元大关，财政收入由3.4亿元到突破百亿元大关，我市用了17年。展望未来，我们用5年的时间就可再造一个钦州，预计2015年工业总产值突破2300亿元，财政收入突破230亿元。我们必须牢牢把握和主动适应这种经济迅速扩张的趋势，紧紧抓住难得的重大机遇和黄金发展期，坚持“以存量换增量、以空间换时间、以资源换产业”的理念，乘势而上，主动作为，继续做大做强经济总量，提升经济质量，增强综合经济实力。

（一）围绕港口国际化现代化，努力形成亿吨吞吐能力。加强基础设施建设，加大港口运营开放力度，全年新增吞吐能力3800万吨以上，吞吐能力超过1亿吨，跻身亿吨能力大港行列。

推进港口深水化大型化建设。建成30万吨级主航道及油码头、大榄坪12#－13#、永鑫10万吨级散货码头、新天德7万吨级散杂货码头等项目，开工建设30万吨级支航道、国投煤炭码头、大榄坪9#－11#泊位、金鼓江石化码头等项目，加快建设金鼓江航道、钦州港三期工程，启动三墩和大环作业区建设，加强锚地规划和建设。

加快完善物流和航运服务体系。围绕建设全国现代物流示范城市和集装箱干线港，积极引进港口、航运、物流大企业进行战略合作，推动港口、物流、园区互动发展。抓好油品、集装箱、煤炭、粮油、石化、汽车、大型综合物流等七大物流系统建设。汽车滚装码头、汽车展示中心、集装箱拆装库、中外运物流仓储等项目建成并投入使用。服务好保税港区和整车进口口岸运营，加快推进保税港区二、三期路网及基础设施建设。开辟新航线，力争新开通钦州至新加坡、马来西亚等东盟国家的国际集装箱班轮航线，加快临港产业发展，大力拓展外贸集装箱货源市场，提升外贸集装箱比重。推进与内陆省市、城市建设“无水港”，加强与集装箱航运公司的合作，完善扶持航运发展政策措施，扩大集装箱运输。利用已建成的国际酒类交易中心、进口汽车检测线及一批仓储物流设施，积极拓展保税物流业务。加大口岸建设资金投入，加快口岸联检楼等项目建设。规划建设航运服务聚集区，优化“大通关”模式，提高口岸服务效率。

加强集疏运能力建设。围绕面向中国－东盟合作区域性国际航运中心、物流中心，逐步完善港口、铁路、公路运输的有效衔接。竣工“四铁两高”，建成南宁至钦州、钦州至北海和钦州至防城港高速铁路、大榄坪至保税港区铁路，钦州至崇左和六景至钦州港高速公路。加快推进龙门跨海大桥、贵港经浦北至合浦高速公路、沿海高速公路改扩建、火车东站综合客运枢纽等项目，开展钦州港集装箱办理站、通用航空基地、平陆运河等项目前期工作。加快港区铁路支线、站场、园区路网建设，推动港区运输网络互通衔接，畅通物流通道。

（二）围绕打造北部湾临海核心工业区，培育形成千亿元产业、千亿元园区和百家亿元企业。坚持临港产业、县域工业联动发展，优化产业结构，营造大、中、小企业集聚发展态势，推动经济质量和效益同步增长，钦州港经济技术开发区成为全区第一个产值超千亿元的国家级园区。

努力壮大临港产业。形成千亿元石化产业。服务好中石油1000万吨炼油厂、东油沥青、玉柴石化等一批石化企业顺产达产，力争中石油含硫原油加工配套工程、中亚石化一期、泰兴石化等项目竣工投产，力促产值超150亿元的玉柴石化二期、580万方油库及产值超50亿元的广州恒诚、南部湾、东油tmp等项目上半年进入全面实质性施工。形成超百亿元的粮油加工、冶金、造纸、能源和现代物流业。力争建成投产汇海100万吨大豆、中粮二期、大洋粮油棕榈油加工；加快进口资源加工区基础设施建设，力争锐丰钒钛铁一期、华汇镍业等项目年内投产，开工建设祥云飞龙二期等项目；加快林浆纸产业园建设，力争金桂一期纸机、钛白粉项目一期建成投产，建成中电投临时供热工程，力争燃煤电厂二期、中电投热电联供、翔盛粘胶纤维等一批项目获核准建设；推进钦州港综合物流加工区、丝茅坪物流中心等物流园区建设，加快建设中国－东盟商贸城、中国—东盟农产品大市场等项目，大力发展现代物流业。

培育高新技术产业。以实施“高新产业培育工程”为载体，完成战略性新兴产业规划，加快40平方公里高新区和综合功能配套区建设，启动高新区二期基础设施建设。提升高新技术产业服务中心科技孵化、创业服务水平。积极扶持和重点培育电子信息、生物技术及新医药、节能环保、新材料、新能源等产业，建成香港清华同方数字电视一期、宇欣电子二期、华成自控等项目，开工建设华泰物联网传感器等项目。大力推进技术改造，保持技改投入稳定增长。

推进县域工业强势发展。制定出台县域企业做大做强激励政策，鼓励通过技改，规下企业冲规上、亿下企业冲亿上企业，力争规模以上县域工业总产值达到420亿元、冲刺500亿元（按旧口径计算），亿元以上产值企业力保95家，冲刺100家。重点服务兴莱鞋业等项目达产，抓好桂金诺、新合力等项目扩建，力促安盛科技、裕源药业等一批项目竣工投产，开工建设青岛九联、天山微电子三期等项目，培育一批上规模、支撑力强的县域骨干企业。实施“中小企业成长工程”，加大对县域中小企业资金、政策等支持，大力发展园区经济，园区产值占县域工业总产值的比重达到60%。

（三）围绕农业增效、农民增收，全力提升特色农业，加快农业产业化进程。以农业科技为先导，以农业增效、农民增收为核心，以实施“特色农业提升工程”为抓手，切实做好“三农”工作，扎实推进新农村建设。

加快提升特色农业。全面实施“特色农业提升工程”，着力培育荔枝、香蕉、奶水牛、大蚝等八大特色产业，通过抓好一批国家级标准果园、钦州“龟谷”、奶水牛规模养殖小区的建设，扩大特色农业标准化示范基地和产业规模，申请特色农产品地理标志登记保护，提升品牌影响力和农业经济效益。继续巩固发展糖料蔗、蔬菜、茶叶、木薯、中草药等已具有相当规模的传统优势产业。切实抓好“米袋子”和“菜篮子”工程，加大常年性无公害蔬菜基地的建设，鼓励冬菜种植，提高我市蔬菜的自给能力；加强耕地保护特别是基本农田保护工作，稳定粮食播种面积，确保粮食安全。

加快推进农业产业化。实施龙头企业、农民专业合作社培育带动工程，开展一村一品的品牌创建行动，加快九联肉食鸡养殖加工一体化等重大农业产业化项目建设，培育一批自治区和市级农业产业化重点龙头企业，新组建农民专业合作社30个，新增“一村一品”专业村8个，新增自治区级以上名优品牌、著名商标2个以上。以钦南台湾农民创业园为平台，深化钦台农业合作，建设高效农业与旅游观光农业一体化示范基地。全力实施农户“万元增收工程”和林下经济“千万林农千元增收工程”，做好农民工转移就业，增加农民生产性收入。落实好农业补贴等惠农政策，确保各类惠农政策资金及时足额兑现到广大农民手中。

加快推进农村基础设施建设。加强社会主义新农村规划，完善农村基础设施建设，开展农村土地综合整治，改善农业和农村生产生活条件，着力增强农业支撑能力。完成30座病险水库除险加固，新建标准海河堤防10公里以上，完成渠道防渗200公里，新增恢复灌溉面积2.5万亩，改善灌溉面积12万亩，治理水土流失面积30平方公里，解决25万农村人口饮水安全问题。修建45个以上建制村沥青（水泥）路，力争建制村通沥青（水泥）路率达到87%以上。积极探索创新“农事村办”等基层工作管理模式。继续实施农业信息化“金农工程”。以“县镇村道路通畅工程三年大会战”为抓手，集中力量推进全市公路路网改造建设，力争用三年时间实现县县通高速公路和一级公路、镇镇通二级公路、村村通硬底化公路，50%的自然村通水泥路。

（四）围绕增强需求动力，扩投资促消费，拉动经济快速发展。

抓项目增投资。举全市之力培育新产业，攻坚大项目，推进大发展。坚持每月联合审批项目机制，完善重大项目领导挂钩、专人跟踪、全程代办和绿色通道制度，加大项目服务效能监察和责任追究力度，推动一批项目集中开竣工，形成新的投资增长点和经济增长点。全年计划组织实施重大项目400个，年度计划投资460亿元，其中新开工重大项目193个，确保全社会固定资产投资完成698亿元、增长25%。

抓市场促消费。全力推动服务业提档升级，成为经济发展的新引擎。积极培育消费增长点。在巩固提高传统服务业的同时，加快发展现代物流、金融保险、信用担保、信息咨询等各类商务服务业，拓展社区商业、物业、家政等便民消费；积极有序发展房地产业，保持房地产市场的持续健康发展。加快建设一批现代服务业集聚区，切实优化商业网点布局，推进中心商业区、特色商业街等商贸网络体系建设，重点依托年年丰广场、金湾步行街提升改造传统商业街区，培育壮大一批品牌龙头商贸企业，积极发展微型企业，增强区域消费集聚效应。积极发展河东新区、钦州港区、灵山县城和浦北新区等片区商业中心。加快发展购物中心、连锁经营、物流配送、电子商务、物流快递等现代商贸流通方式。继续做好“万村千乡市场工程”等促消费活动，降低流通成本，扩大城乡居民消费。

（五）围绕增强城市文化软实力，挖掘城市的历史文化内涵，打造旅游文化品牌。

推进文化大发展大繁荣。深入实施“文化惠民工程”，加快公共文化服务体系建设，开工建设北部湾博物馆、市群众艺术馆、市图书馆、刘永福纪念馆、文化科技活动中心以及一批街道、乡镇、社区、村文化活动中心；完善文化产业发展扶持政策，扶持壮大坭兴陶、手工艺编织等传统文化产业，培育发展文化创意、动漫游戏等新兴文化产业，启动建设坭兴陶文化创意产业园和坭兴陶博物馆；加快发展广电影视事业，继续实施农村、开发区、校园电影公益性放映工程、村村通广播电视工程；加强与东盟国家的文化产业交流合作，提高文化产业的外向度。加大“扫黄打非”工作力度，加强文化遗产保护，加快发展新闻出版事业，规范和繁荣文化市场。

推进特色旅游业大发展。依托山海旅游资源优势和南宁至钦州城际快铁通车的交通优势、南宁与钦州同城化的优势，着力打造旅游精品项目。利用茅尾海国家级海洋公园的品牌，高标准高质量开展茅尾海综合整治，保护开发好茅尾海资源；加快三娘湾主题乐园、八寨沟森林旅游休闲区、沙井岛美食城、大芦村民俗旅游区、五皇山国家地质公园等旅游景点基础设施建设。大力挖掘刘冯、坭兴陶等人文历史旅游资源，积极筹划坭兴陶创意大赛和第二届蚝情美食节，继续办好三娘湾观潮节、灵山荔枝节、浦北香蕉节，培育我市的节庆文化旅游，大力发展滨海风情游、山水田园休闲游等特色生态旅游。加快北部湾国际大酒店等重点项目建设，培育“绿色饭店”、“绿色餐饮企业”。加强旅游宣传促销，拓展客源市场，增强钦州旅游的影响力。

四、千方百计抓住城镇化进程加快的机遇，加大城乡统筹力度，努力提升城镇化水平

目前，全国、全区的城镇化进程呈加快趋势，2011年全国、全区城镇化率已分别达到50%和42%，“十二五”期间，广西城镇化率将保持每年2个百分点的增速，2015年将达到50%。而我市2011年仅达到37.5%，要赶上和超过全国和全区水平，“十二五”期间必须以每年3个百分点的速度推进。要按照“东进南拓、向海发展”的空间布局，完善城市规划体系，以滨海新城建设为重点，全力扩大城市规模，完善城市功能，打造区域竞争新优势。

拓展城市规模。以白石湖片区、辣椒槌片区、沙井岛片区开发为重点，推动滨海新城建设整体出形象。白石湖片区要建成北部湾国际大酒店、滨湖路、安州大道等项目，开工建设北美商贸城、五洲大厦、高投大厦等；辣椒槌片区要加快建设东盟生态园、茅尾海综合整治一期工程等项目，开工建设皇冠假日酒店；沙井岛片区要建成钻石海岸国际海鲜城、沿海高速公路茅尾海出口，加快建设北部湾大学滨海校区等项目。拓展河东新区。完善河东区路网和配套建设，力促一批房地产项目开工和加快建成，扩大建成区规模。加快钦州港行政商务中心建设，力促联检部门办公用房、农民安置房及学校、医院等配套设施尽快建成投入使用。稳步推进旧城改造，启动一马路至五马路的老街区保护和改造工作，大力推进千年古陶城建设。

完善城市功能。加快建设“一环六横六纵七通道”城市主干路网，建成扬帆大道南延长线，加快建设北部湾大道二期、环城东路、环城北路，开工建设钦江五桥，做好环城西路南段的前期工作，逐步形成城市环路。加快建设公共服务设施，建成河东污水处理厂、大榄坪污水处理厂以及汽车北站、油麻角公交综合站场等一批公共站场和便民候车亭；加快市第三水厂、大榄坪水厂及河东区、河西区供水管网工程和城市污水管网建设，开工建设垃圾焚烧发电厂一期工程。

提升城市形象。深入实施“园林生活十年计划”，完善城市绿地系统，建成白石湖公园、和谐塔公园、市行政中心北面公园、黎合江公园等一批城市公园和公共绿地，启动改造和扩建中山公园，加快钦江综合治理，提高城市园林绿化水平。积极推进“数字城管”建设，进一步理顺城市管理体制，实现城市管理重心下移，形成责权明确、务实高效、运行有序的城市管理格局。推行精细化市政管理，深入实施“城乡清洁工程”和“城乡风貌改造”，开展以治理“五乱”为重点的市容环境整治活动，促进城市净化、美化、亮化，改善城市景观，确保成功创建自治区文明城，全面启动国家园林城市、国家卫生城市的创建工作。

加强重点镇建设。强化城乡一体化规划和建设，统筹城乡协调发展。把城镇建设与工业集中区建设、生态环境建设、农业产业化经营、发展城镇经济紧密结合起来，扩大城镇规模，完善城镇功能，不断增强城镇对农村劳动力转移的接纳能力和对农村经济的带动作用。大力推进县城和重点镇建设，推进一批镇改街道办事处和村委会改居委会工作，逐步形成以市区为主体，以重点镇为依托，城乡协调发展的新型城镇化发展格局，加速城镇化进程。

五、千方百计抓住区域发展呈现产业、要素加快集聚的机遇，全力打造区域合作新高地

随着保税港区、整车进口口岸、中马钦州产业园区、国家级经济技术开发区等特殊政策和平台落户钦州，外部交通环境的不断改善以及重大产业项目的带动效应，产业、项目、资金、技术等生产要素将加快向我市集聚。我们要顺势而为，加强区域合作，充分利用国内外两个市场两种资源，形成产业集群效应，谋求在合作中实现互利共赢。

（一）围绕打造国际区域合作新高地，拓展全方位的合作与交流。按照“提升东盟、巩固港澳台、突破日韩、发展欧美”的思路，发挥区位和政策优势，加快园区发展，强化招商引资，形成区域合作新优势。

夯实合作发展平台。重点推进四大园区建设。石化产业园要服务好企业入园，加快延伸石化产业链，实现千亿元产能目标，力争升级为国家级石化园区。中马钦州产业园区要尽快完成各项规划编制和启动区征地搬迁，加快建设“一街两道一湾一门一楼一坝”以及管网、供水、供电、排污等设施，积极研究合作机制和开发模式，加大招商力度，力争一批产业项目落户园区。装备制造产业园要围绕把装备制造业培育成第二个千亿元产业的目标，加快推进7.3平方公里装备制造产业园海域吹填、水电路等基础设施建设，积极争取国家和自治区在政策、资金、用地用海等方面的扶持，全力争取汽车、修造船、飞机制造、港口机械项目落户建设。河东工业园区要争取成功申报自治区级高新区，大力培育发展战略性新兴产业，确保清华同方、宇欣电子、骏升科技等项目年内建成投产，推动力顺二期等项目动工建设；启动园区二期开发建设，力争开工一批生物制药等项目。改革创新华侨农林场发展模式，开发建设北部湾华侨投资区。继续推动湖南临港产业园等一批跨区域合作园区加快建设。

扩大交流合作。依托钦州拥有的政策平台优势，加强在港口、产业、城市建设、园区建设、贸易便利化等方面进行全方位合作。大力发展外向型经济，推进石化、造纸、电子、粮油等一批出口商品基地建设，提升外经贸水平。引导扶持大型企业在钦州设立具有自主进出口经营权的分支机构，继续争取广西中石油国际事业公司原油非国营贸易进口资格和配额，扩大对外贸易规模。积极实施“走出去”战略，引导和鼓励有条件的企业到境外投资和经营，开拓东盟和国际市场。

全力招大引强。把招商引资摆上更加突出的位置，坚持政府招商与社会招商齐头并进，配强力量，创新方式，不断提升招商实效。继续发挥专业招商团队的作用，围绕培育千亿元产业和百亿元产业，提高项目策划包装质量。瞄准世界500强、中国100强和行业前10强的大企业、大集团，加强中马钦州产业园区、石化产业园、综合物流加工区、钦州保税港区等园区招商推介，力争在汽车装备制造、修造船、芳烃等重大产业项目引进取得重大突破。县区工业集中区重点加大对中小企业的招商引资力度，确保大项目顶天立地，中小企业铺天盖地。加快项目审批和要素协调，确保重大项目快落户、早开工、快投产。完善招商引资考评机制，开展“招商引资大兑现”活动，提高合同履约率、资金到位率、项目开竣工率。

（二）围绕激发体制机制活力，进一步加大改革创新力度。

扎实推进改革创新。争取设立北部湾产业投资基金，继续扩大发行企业债券、信托融资等。积极利用bt方式融资，培育2－3家企业上市直接融资，推动发行中小企业集合债券，完善市中小企业信用担保服务平台，新增一批小额贷款公司。加强和改进土地资产经营管理，严格节约集约用地，提高土地利用效率，继续完善“征、拆、储、建、安、保”六位一体征拆模式，推进城乡建设用地增减挂钩、土地“征转分离”和海域使用权招拍挂试点。实施领军型人才引进计划，完善政府雇员制度。创新人才工作机制，加快北部湾国际人才创业基地建设。创新园区管理体制，增强园区自主权，构建高效的运转机制。制订吸引民间资本产业指导目录及政策措施，大力扶持发展中小微型企业。完善项目管理机制，制订出台重大产业项目预评估管理办法、政府投资项目后评价管理办法。深化财政改革，强化预算管理标准化、精细化，统筹财政收支，优化支出结构，防御政府债务风险；设立第三产业发展专项资金，涵养地方税源，增加可用财力。稳妥推进医药卫生、教育、文化、社会管理、事业单位、华侨农林场、国有企业等改革。

支持科技创新。充分发挥科技创新的引领和支撑作用，加强高新技术企业培育和认定工作，加强工程技术研究中心、重点实验室等技术创新载体建设，培育拥有自主知识产权的创新型企业，增强自主创新能力。加强高技能人才队伍建设，积极引进高层次人才，以人才工作机制创新激发各类人才的创造活力和创业热情。加强科学普及和知识产权保护。加强科技兴农，提升农业综合生产能力。

六、主动适应经济与社会协调发展的新要求，全力改善和保障民生，促进社会和谐稳定

注重强化民生保障。深入实施“三业”行动富民工程，建立健全劳务合作、用工对接等机制，加强职业技能培训，突出抓好农民工、被征地农民、失海渔民、城镇零就业家庭等特殊群体的就业帮扶，重点扶持中专技校毕业生、城镇失业人员、返乡农民工、库区移民、被征地拆迁对象、城乡退役军人等人群创业，以创业带动就业，实现就业、创业倍增效应。积极稳妥地推进农村社会养老保险试点工作，建立健全社会保险、社会救助、社会福利等社会保障体系。推进社会养老服务体系建设，不断完善孤儿保障制度。实现城镇各类就业人员平等享有社会保险，基本养老保险、医疗保险、失业保险、工伤保险、生育保险参保覆盖面进一步扩大。

注重解决民生难题。围绕与群众生产生活密切相关的问题，投资34.27亿元，继续开展为民办实事工程。加快保障性安居工程建设，建立健全分配和运营监管机制，建成廉租房1600套、经济适用房1100套、公租房2000套。实施新一轮扶贫攻坚战，继续推进整村扶贫工程，每个县区开展一个以上的贫困村整体帮扶试点；继续实施农村危房改造，切实改善农村人居环境；完善家庭经济困难学生资助体系，确保每个学生不因家庭经济困难而失学。推进农村教师周转房建设，努力改善农村教师工作生活条件。

注重社会全面发展。坚持教育优先发展。加快北部湾大学建设，8万平方米教学实验用房全面封顶；加快发展职业教育，抓好市、灵山、浦北职教中心办学；进一步优化中小学校布局结构，提高义务教育巩固率，实施高中课程改革；加强特殊教育；推进中小学校舍安全工程建设，加快推进市四中重建、沙埠中学扩建等项目，开工建设浦北金浦中学、市实验小学白石湖校区、市二中附属幼儿园等一批项目。加快发展卫生事业。加快完善全民医保制度，城镇职工、居民医保和新农合参保率继续提高并稳定在95%以上。巩固扩大国家基本药物制度实施范围和基层综合改革。加快市级医院建设，完成市第一人民医院扩建工程、市第二人民医院综合楼、精神病院综合楼。健全基层医疗卫生服务体系，推进完善县区人民医院和社区卫生服务中心、乡镇卫生院、村卫生室等标准化建设。做好基层医疗机构人员培养、培训，加强村级医疗队伍建设，加快清理化解基层医疗卫生机构债务。继续实施重大公共卫生服务项目，不断提高公共卫生服务均等化水平。稳步推进县级公立医院的管理体制、补偿机制、运行机制等方面的综合改革。统筹工业发展和节能减排，大力发展低碳型循环经济产业，加快节能减排工程建设，淘汰落后产能，强化节能减排目标责任，抓好大气污染联防联控工作，促进绿色发展。全面推进诚信计生，提升人口和计生服务质量，稳定低生育水平，提高人口综合素质。加强防灾减灾体系建设，普及提高综合应急处置和救援能力。落实各项优抚安置政策，支持部队和国防建设，深入开展双拥共建活动，巩固和发展军政军民团结。加强民族工作，巩固和发展平等、团结、互助、和谐的民族关系。继续做好工会、共青团、妇联、外事、新闻出版、科普、侨务、统计、海防、地震、宗教、气象、水文、档案、地方志、老龄和残疾等工作。

注重维护社会稳定。加强和创新社会管理，深入开展“携手共创平安和谐家园”活动。加强农村基层组织建设，完善社会矛盾纠纷大调解机制，努力化解社会矛盾。加强社会治安防控体系建设，着力解决动态环境下的治安防控问题。加快“天网”三期建设，力争实现市、县区、重点镇三级联网。加强政法基础设施建设，推进市、县公检法司业务技术用房和戒毒所、拘留所、看守所等监管场所建设。推进市、县地震避难所、应急救灾所等防灾减灾项目建设。强化“安全发展”理念，突出抓好烟花爆竹、危险化学品、港口码头、非煤矿山等重点行业和领域的安全监管，确保安全生产形势平稳。加强安全生产隐患排查整治，确保企业生产、食品药品、校园校车、交通和消防等安全。健全食品药品监管体系，强化监管，切实保障群众饮食、用药安全。

各位代表，经济社会协调发展，是我们建设更高水平全面小康社会的必然选择。我们将坚持经济越发展越要重视社会建设和民生改善，让发展成果惠泽全市人民，让人民生活更具品质，让社会更加充满活力、更加和谐稳定！

七、主动适应经济社会转型发展的新要求，加强自身建设，建设人民满意政府

在历届政府打下的良好基础上，钦州已成为广西发展最快、后劲最足、最令人瞩目的城市之一。如何在更加激烈的区域经济竞争中抢占制高点、增创新优势、保持好态势，如何有效应对和解决社会转型时期出现的新矛盾、新问题，这是对政府工作的新考验。我们务必增强忧患意识、紧迫意识，切不可坐井观天、盲目乐观；务必主动适应形势发展的新要求、顺应人民群众的新期盼，切不可精神懈怠、作风漂浮。要始终把加强政府自身建设摆在突出位置，努力建设人民满意政府，更好地为改革发展大局服务、为人民群众造福。

更加注重开拓创新，拓宽解决问题的思维和途径。深入开展“解放思想、赶超跨越”大讨论活动，以强烈的创新意识，推动经济的大发展。要破除小进即安的思想，增强开拓创新、抢抓机遇的勇气，敏锐地发现机遇、抓住机遇、用好机遇。要破除按部就班的思想，增强敢闯敢拼、勇创大业的士气，敢于超常规，勇于迈大步，善于办大事。要破除得过且过的思想，增强知难而上、奋勇争先的锐气，围绕全市的中心工作，找准工作的切入点和突破口，攻坚克难，营造奋发向上、勇创一流的发展环境。

更加注重依法行政，增强责任意识和法制观念。健全科学民主决策制度，坚决贯彻落实市委各项重大决策部署，坚持重大事项向人大报告和政协通报制度，主动接受人大依法监督、政协民主监督以及司法监督和社会监督，加强政府与人大代表、政协委员的联系。认真开展“六五”普法教育，不断增强全民法制意识。完善重大决策程序，对涉及经济社会发展大局的重大事项，广泛征求意见，提高决策科学性。

更加注重行政效能，提高政府执行力与公信力。深入开展“好干部善执行”主题活动。深化行政审批制度改革，强化“马上办”意识，开展“行政审批提速工程”，创新行政审批方式，提高审批效率。推进政务服务和政务公开向基层延伸，完善平衡计分卡绩效考评制度，继续推行“阳光和效能重点建设岗位”制度，开展扩权强县工作。畅通民意诉求渠道，完善领导下访制度，继续办好“12345”市长热线，提高为民办事实效。加强电子政务建设，完善政务公开制度，扩大群众参与度，不断提高政府工作透明度，保障人民群众的知情权、参与权和监督权，提高政府公信力。

更加注重队伍建设，强化节俭约束与廉政防线。大力弘扬高效务实的工作作风，以只争朝夕的精神、坚韧不拔的干劲，深入一线抓项目、深入实际破难题、深入群众解民忧，确保各项工作快速推进、目标任务高效落实。深入开展政风、行风评议活动和纠风治乱工作，着力解决群众反映强烈的问题。厉行节约，强化财政预算约束，严控行政开支，降低公务成本。认真落实党风廉政建设责任制，严格执行廉洁从政各项规定，强化领导干部自律教育，深化推进惩防体系建设。规范公共资源交易平台建设，加强对工程招投标和政府采购等公共资源交易的监督管理。开展“审计整改年”活动，加大对重点领域、重大工程、重要环节的审计和监察力度，努力从源头上预防腐败。严肃查处违法违纪案件，严厉惩治腐败。

各位代表，挑战激发斗志，机遇催人奋进。让我们在市委的坚强领导下，大力弘扬“自强实干、融和共赢”的新时期钦州精神，脚踏实地、勇于开拓、善于创新，奋力开创我市科学发展新局面，以优异成绩迎接党的十八大胜利召开！
